# Genetic and metabolic links between the murine microbiome and memory

**DOI:** 10.1186/s40168-020-00817-w

**Published:** 2020-04-16

**Authors:** Jian-Hua Mao, Young-Mo Kim, Yan-Xia Zhou, Dehong Hu, Chenhan Zhong, Hang Chang, Colin J. Brislawn, Sarah Fansler, Sasha Langley, Yunshan Wang, B. Y. Loulou Peisl, Susan E. Celniker, David W. Threadgill, Paul Wilmes, Galya Orr, Thomas O. Metz, Janet K. Jansson, Antoine M. Snijders

**Affiliations:** 1 Biological Systems and Engineering Division, Lawrence Berkeley National Laboratory, Berkeley, CA 94720 USA; 2 Earth and Biological Sciences Directorate, Pacific Northwest National Laboratory, Richland, WA USA; 3 Marine College, Shandong University, Weihai, 264209 China; 4 Department of Clinical Laboratory, The Second Hospital of Shandong University, Jinan, 250033 Shandong China; 5 Luxembourg Centre for Systems Biomedicine, University of Luxembourg, 7, Avenue des Hauts Fourneaux, L-4362 Esch-sur-Alzette, Luxembourg; 6 Department of Veterinary Pathobiology, A&M University, College Station, Texas, USA; 7 Department of Molecular and Cellular Medicine Texas, A&M University, College Station, Texas, USA

**Keywords:** Collaborative Cross mouse model, Memory, Gut–brain axis, Lactobacillus, Germ-free, Metabolites, Lactate, GABA

## Abstract

**Background:**

Recent evidence has linked the gut microbiome to host behavior via the gut–brain axis [1–3]; however, the underlying mechanisms remain unexplored. Here, we determined the links between host genetics, the gut microbiome and memory using the genetically defined Collaborative Cross (CC) mouse cohort, complemented with microbiome and metabolomic analyses in conventional and germ-free (GF) mice.

**Results:**

A genome-wide association analysis (GWAS) identified 715 of 76,080 single-nucleotide polymorphisms (SNPs) that were significantly associated with short-term memory using the passive avoidance model. The identified SNPs were enriched in genes known to be involved in learning and memory functions. By 16S rRNA gene sequencing of the gut microbial community in the same CC cohort, we identified specific microorganisms that were significantly correlated with longer latencies in our retention test, including a positive correlation with *Lactobacillus*. Inoculation of GF mice with individual species of *Lactobacillus* (*L. reuteri* F275*, L. plantarum* BDGP2 or *L. brevis* BDGP6) resulted in significantly improved memory compared to uninoculated or *E. coli* DH10B inoculated controls. Untargeted metabolomics analysis revealed significantly higher levels of several metabolites, including lactate, in the stools of *Lactobacillus*-colonized mice, when compared to GF control mice. Moreover, we demonstrate that dietary lactate treatment alone boosted memory in conventional mice. Mechanistically, we show that both inoculation with *Lactobacillus* or lactate treatment significantly increased the levels of the neurotransmitter, gamma-aminobutyric acid (GABA), in the hippocampus of the mice.

**Conclusion:**

Together, this study provides new evidence for a link between *Lactobacillus* and memory and our results open possible new avenues for treating memory impairment disorders using specific gut microbial inoculants and/or metabolites.

Video Abstract

## Background

Specific members of the gut microbiome have been linked to host health and behavior [4]. Intriguingly, probiotics comprised of different *Lactobacillus* and/or *Bifidobacterium* strains have been shown to impact behavior in mice, including reduction of symptoms linked to anxiety [5–7] and improvement of memory [8, 9]. Administration of probiotic strains specifically results in an improvement in memory of objects and object location [8–11], but not object temporal order memory [8].

Metabolic clues to memory enhancement have been found by analyzing metabolic signatures in the brains of mice following administration with specific *Lactobacillus* strains. Increased levels of GABA in the brain is linked to improved working memory and novel object recognition [12, 13]. Mice fed with *L. rhamnosus* JB-1 had increased mRNA expression of the GABA receptor [5], and increased metabolic levels of GABA in the hippocampus [14]. Increased levels of GABA in the brain could also be due to increased production of GABA by gut bacteria [15, 16]. However, the metabolic mediator(s), if any, between the gut and the brain remain unknown. Recently, O'Hagan et al. (2017) found increased levels of lactate in the brains of mice that were fed supplements containing a mixture of lactobacilli and bifidobacteria: *L. acidophilus* CUL60, *L. acidophilus* CUL21, *B. bifidum* CUL20 and *B. lactis* CUL34) [8]. Together, these studies suggest a link between specific metabolites produced by lactobacilli and memory of the host via the gut–brain axis that remains to be further explored and validated.

The complex interplay between host genetics, environment, and lifestyle factors and the gut microbiome make studying the role of the microbiome on memory potential difficult in human populations. Model systems can help overcome this barrier and offer many advantages for the study of the genetic basis of complex phenotypes. The “Collaborative Cross” (CC) is a population-based mouse model system with genetic and phenotypic diversity on par with the human population [17]. The CC, which captures nearly 90% of the known variation present in laboratory mice, was established by combining the genomes of eight diverse founder strains (A/J, C57BL/6J, 129S1/SvImJ, NOD/LtJ, NZO/HlLtJ, CAST/EiJ, PWK/PhJ, and WSB/EiJ). The advantage of the CC is that genetic and environmental components of risk can be specified and tightly controlled allowing for a comprehensive analysis of the role of host genetics and the microbiome on memory. In this study for the first time, we performed an unbiased genetic screen using CC mice to identify host genetic and microbiome components that are associated with memory potential. Subsequently, we used this information to focus on specific strains that were correlated with memory in the CC mouse cohort and to evaluate their metabolic profiles in a gnotobiotic mouse system in order to better understand the metabolic mechanisms underlying memory improvement.

## Results

**Fig. 1:**
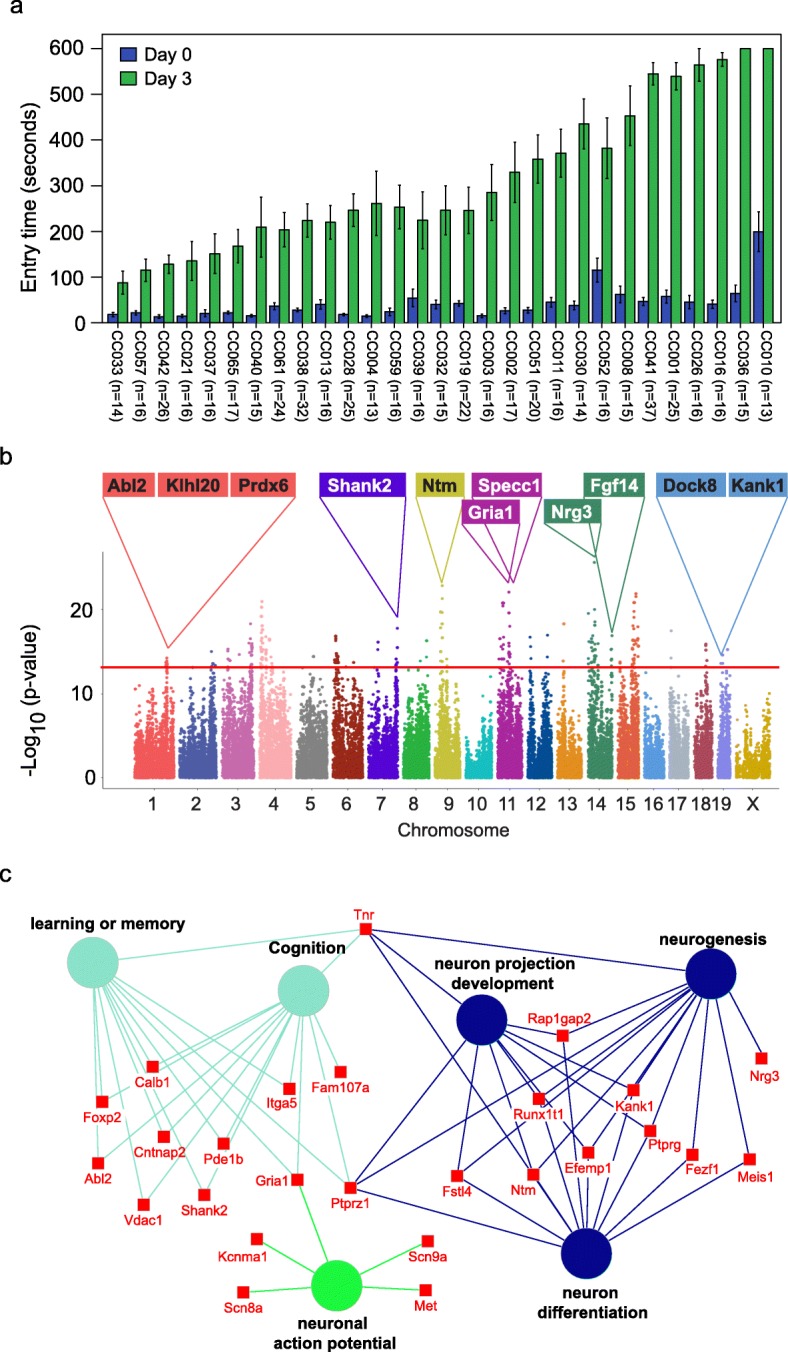
Identification of genetic variations and candidate genes associated with memory in CC mice. **a** Variations in memory across CC strains. Memory was assessed using passive avoidance, a fear-motivated test. The memory test is based on latency of entry into a compartment where 3 days earlier a mild foot shock (0.3 mA for 5 s) was experienced. Entry into the shock compartment on Day 0 is shown in blue, whereas entry 3 days after the foot shock is shown in green (Day 3). Mice with good memory avoided entering the chamber on Day 3, whereas mice with poor memory entered the chamber. Error bars indicate mean ± SEM. **b** Manhattan plot of the GWAS analysis for memory in CC mice (*n* = 535 mice). The – log_10_(*P* value) is shown for 76,080 SNPs ordered based on genomic position. The horizontal red line indicates the QTL significance threshold at − log_10_(*P* value) = 12. Candidate genes previously associated with memory, cognition, or other neurodevelopmental processes located in representative QTL are listed above the plot. **c** Gene ontology (GO) analysis of genes identified in QTL associated with memory potential in Fig. 1b (*n* = 535 mice). Genetic loci are significantly enriched for genes implicated in learning or memory, cognition, neuron projection development, neurogenesis, neuron differentiation, and neuronal action potential

We assessed memory using passive avoidance, a fear-motivated test to assess memory-dependent hippocampal function [18, 19], in 535 mice from 29 Collaborative Cross (CC) strains (Table S1). The passive avoidance memory test is based on latency of entry into a compartment where three days earlier, a mild foot shock (0.3 mA for 5 s) was experienced. Mice with good memory avoided entering the chamber where they had previously been exposed to the shock, whereas mice with poor memory entered the chamber. There were significant and reproducible variations in memory potentials across the different CC strains (Fig. 1a). The latency in entry time on the testing day ranged from 87.9 to 600 s (Fig. 1a). Mice from two strains (CC036 and CC010) never entered the chamber within the 600 s assay time. We observed sex differences in memory potential in two of the strains (CC019 and CC032) where memory potential was higher in male mice, whereas no significant sex difference was observed for any of the other strains (Figure S1).

The reproducible variation in memory potential across the CC cohort suggests that host genetics plays an important role in memory. To identify the potential genetic variations that contribute to memory, we performed GWAS with 76080 SNPs across 29 CC strains. We identified 715 SNPs significantly associated with memory (*p* value < 10^−12^), corresponding to 222 annotated genes (Fig. 1b, Table S2). Gene set enrichment analysis revealed that the 222 genes were significantly enriched in biological processes related to learning or memory (*p* = 1.87E–5), neuron cellular component (*p* = 3.97E–9), and abnormal learning/memory/conditioning phenotypes (*p* = 1.03E–4; Fig. 1c, Table S3) [20]. In addition to 71 genes known to be associated with memory and learning, our screen also identified 135 genes not previously associated with memory including 65 genes that show expression in the brain based on in situ hybridization data from the Mouse Brain Atlas (Allen Brain Atlas; Table S4). The spatial gene expression data suggest that these 65 genes may play a role in memory.

**Fig. 2:**
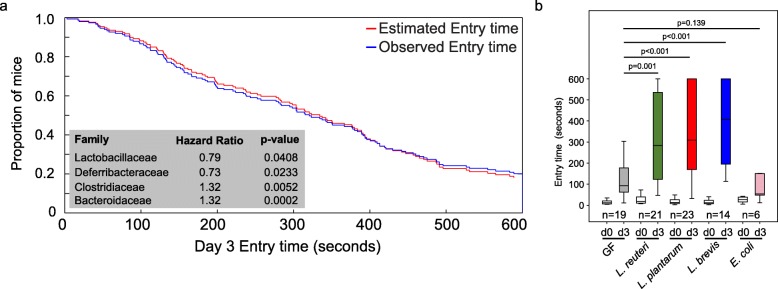
The impact of *Lactobacillus* or lactate treatment on memory. **a** Identification of microbes associated with memory in CC mice by multivariate Cox regression analysis. **b** Inoculation of GF mice with individual species of *Lactobacillus* (*L. reuteri* F275*, L. brevis* BDGP6 or *L. plantarum* BDGP2) resulted in significantly improved memory compared to uninoculated or *E. coli* inoculated controls. Error bars indicate mean ± SEM

Previously, we demonstrated natural host variation in the gut microbiome composition across CC mice [21]. To determine links between specific members of the gut microbiome and memory, we correlated 16S rRNA gene sequence data to memory of individual CC strains. Sequence reads were mapped to 5761 OTUs corresponding to 72 bacterial families (Table S5). After filtering OTUs to those with > 100 reads 41 families remained. Four families (*Lactobacillaceae*, *Deferribacteraceae*, *Bacteroidaceae*, and *Clostridiaceae*) were significantly correlated with memory based on multivariate Cox regression analysis (*p* < 0.05; Fig. 2a). The hazard ratio (HR) indicated that higher relative abundances of *Lactobacillaceae* (specifically *L. reuteri;* HR = 0.79) and *Deferribacteraceae* (HR = 0.73) and lower relative abundances of *Bacteroidaceae* (HR = 1.32) and *Clostridaceae* (HR = 1.32) predicted improved memory potential (Fig. 2a). Because other *Lactobacillus* strains have previously been implicated in improving memory in mice, humans, and rats: e.g., *L. rhamnosus* JB-1 in mice [5], *L. casei* LC122 in aged mice [22], *L. helveticus* ROO52 in humans [6], and *L. acidophilus* strains CUL60 and CUL21 (in combination with bifidobacteria) in rats [8]. We focused our remaining studies on *Lactobacillus*, in particular *L. reuteri* that was identified in this study.

To investigate the impact of *Lactobacillus* on memory, we colonized separate cohorts of germ-free (GF) mice by oral gavage with *L. reuteri* F275*.* This species has an exact 16S rRNA gene sequence match to the OTU that was significantly correlated with improved memory. In addition, we included two other *Lactobacillus* species for comparison *(L. plantarum* BDGP2 and *L. brevis* BDGP6*)*. Within our three inoculated groups, we validated the presence of the individual *Lactobacillus* OTUs, each of which was dominant in a single treatment group and could be traced to our inoculated species (Figure S2). Memory was assessed using the same passive avoidance test in the *Lactobacillus* inoculated mice and compared to GF mice of the same genetic background. *Eschericia coli* DH10B inoculated mice were included as a negative control. Our data showed that all of the *Lactobacillus* mono-associated mice showed a significant improvement in memory compared to GF mice (*p* < 0.001; Fig. 2b). In contrast, we observed no memory improvement following application of *Escherichia coli* DH10B (Fig. 2b).

**Fig. 3:**
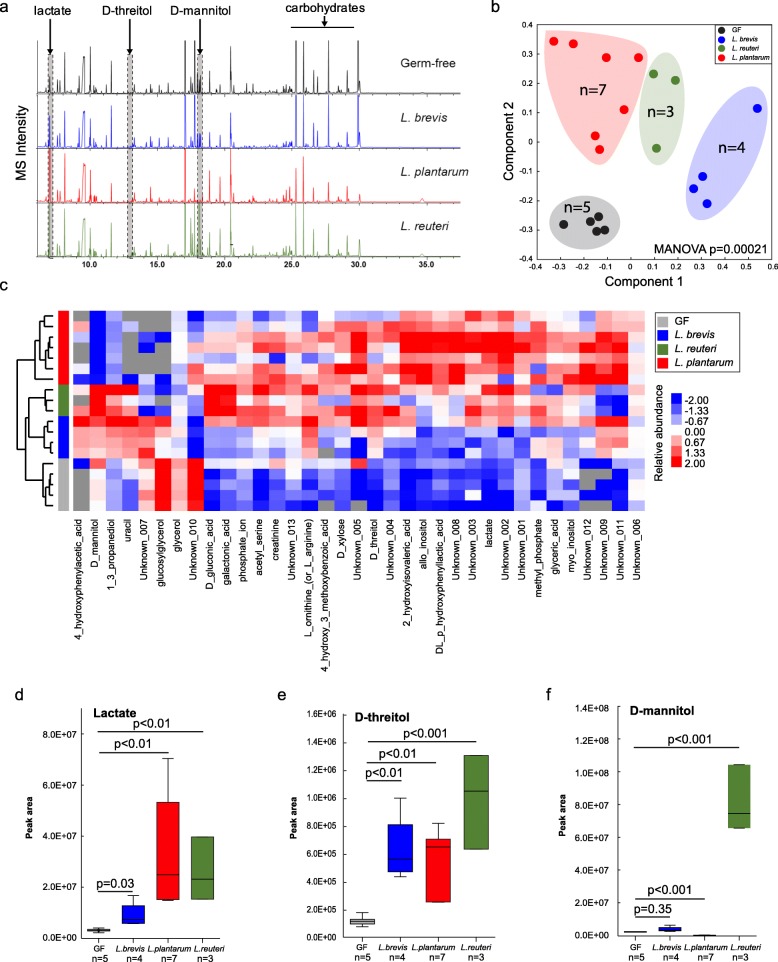
Metabolomics analysis of fecal samples from *Lactobacillus*-colonized and germ-free mice. **a** Representative GC–MS chromatograms of metabolite profiles in germ-free and *Lactobacillus*-colonized mouse fecal samples. Each chromatogram is a representative mass spectrometry profile from one cage of mice (4 mice/cage). **b** PCoA of metabolite profiles were measured in fecal samples. **c** Heatmap of metabolites differentiated between *Lactobacillus*-colonized and germ-free mice. **d–f** Relative abundance of select metabolites in fecal samples from individual mice. Error bars indicate mean ± SEM

To identify metabolites produced by lactobacilli that are candidates for microbiome mediated memory enhancement, we assessed the metabolome in fecal samples collected from GF and mice mono-associated with one of each of the three *Lactobacillus* species: *L. reuteri* F275, *L. plantarum* BDGP2 or *L. brevis* BDGP6 (Fig. 3a, Figure S3, Table S6). The LLE (local-linear embedding analysis) plot shows that the metabolite composition in fecal samples from the *Lactobacillus*-colonized mice and GF mice were distinct (*p* = 0.00021; Fig. 3b,c), demonstrating that *Lactobacillus* inoculation significantly affected the gut metabolome. Based on gas chromatography–mass spectrometry (GC–MS) peak intensities, lactate and threitol were consistently higher in stool samples collected from mice that were colonized with any of the three strains (*p* < 0.01; Fig. 3d,e; Fig. S3), whereas some strain-specific differences in gut metabolites were seen, such as D-mannitol that was only higher in *L. reuteri* inoculated samples (*p* < 0.001; Fig. 3f; Fig. S3). Subsequent injection of mice with mannitol did not enhance memory (data not shown). Other examples of metabolites showing highed levels in fecal samples from *Lactobacillus* inoculated mice include: galactonic acid (*L. reuteri*); D-xylose, glyceric acid, and methyl phosphate (*L. plantarum*); and uracil (*L. brevis*; Fig. S3A). Also, many of the GC–MS peaks corresponding to “carbohydrates”, presumably from the mouse chow, were lower in intensity in stool samples colonized with the *Lactobacillus* species suggesting that the components were being degraded by the inoculants (Fig. 3a).

To determine which metabolites could be possible mediators of the memory response, we also identified specific metabolites that were significantly higher in plasma and brain homogenates from the colonized mice compared to GF mice (Fig. S3). Fewer metabolite differences were found between species in plasma and brain, with the exception of higher plasma levels of arabitol, citric acid, glucose and L-tryptophan (*L. plantarum*; Fig. S3B). In the brain homogenates, species-specific differences included significantly higher levels of D-malic acid, dehydroascorbic acid, GABA, lactate, methyl phosphate, myo-inositol, and scyllo-inositol (*L. plantarum*); and glycine (*L. reuteri*; Fig. S3C). Examples of brain metabolites that were significantly higher in *L. reuteri* or *L. plantarum* inoculated mice compared to GF controls include glycerol, L-glutamic acid, L-serine, and N-acetyl-L-aspartic acid (Fig. S3C). Also, for both *L. plantarum* and *L. brevis* inoculated mice, 2,5-dihydroxypyrazine and citric acid were significantly higher in brain homogenates compared to GF controls (Fig. S3C).

Because we found an improved memory response with each of the three *Lactobacillus* species, we focused on fecal metabolites that were consistently higher across all three species compared to GF controls (Fig. S3), these included statistically (*p* < 0.05) higher levels of lactate (Fig. 3d), D-threitol (Fig. 3e), 2-hydoxyisovaleric acid, and acetyl-serine (Figure S3A). Interestingly, glycerol was significantly lower in fecal samples from mice inoculated with the three *Lactobacillus* species compared to GF controls (Fig. S3A). For the plasma samples, 1,5-anhydrohexitol, carbonate ion, pyruvic acid, and xylitol were significantly higher in all of the *Lactobacillus* inoculated mice compared to the GF controls (Fig. S3B). However, none of the identified metabolites were significantly higher in brain homogenates of *Lactobacillus* inoculated mice when compared to GF controls (Fig. S3C). Lactate did, however, have a trend towards higher levels in both plasma and brain, and was only significantly higher than GF controls (*p* < 0.05) in the brain samples from *L. plantarum* inoculated mice (Fig. S3C).

**Fig. 4:**
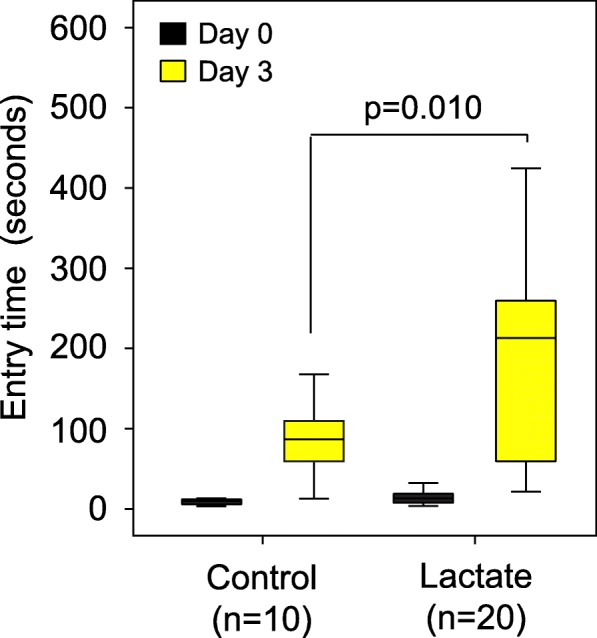
Dietary lactate treatment alone significantly boosted memory in CC042 mice. CC042 mice were treated with lactate through drinking water (*n* = 20; 0.5 g lactate/100 ml water) for 5 weeks or control (*n* = 20). Memory was assessed using passive avoidance. Dietary lactate treatment significantly improved the average retention latency period of the CC042 mice from 92 s to 210 s. Error bars indicate mean ± SEM

Based on these metabolite data and other studies of the role of lactate in memory formation [23], we hypothesized that lactate could be a mediator of the improved memory response in our trials because lactate is commonly produced by all lactobacilli. Also, lactate was recently shown to be higher in brain samples from rats that consumed a dietary supplement containing *Lactobacillus* and *Bifidobacterium* strains, and the rats had an improved memory as a result [8]. Therefore, we conducted an experiment to determine whether mice treated with dietary lactate had improved memory. CC042 mice, which have a relatively poor memory in the passive avoidance memory test (Fig. 1a), were treated with lactate through drinking water (0.5 g lactate/100 ml water) for 5 weeks. We found that dietary lactate treatment improved the average retention latency period of the CC042 mice from 92 s to 210 s (*p* = 0.01; Fig. 4).

**Fig. 5:**
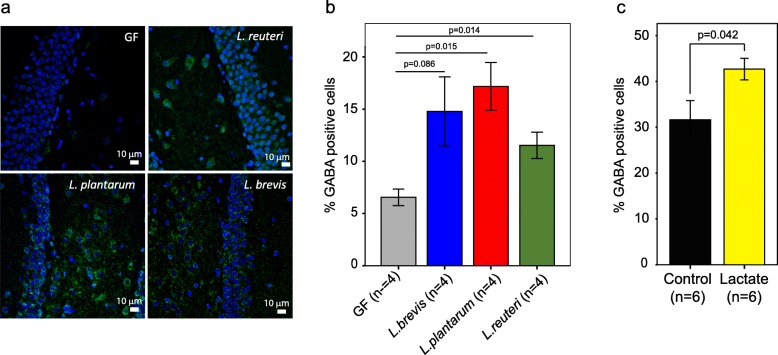
Influence of *Lactobacillus* inoculation or lactate treatment on the levels of the neurotransmitter, gamma-aminobutyric acid (GABA), in the hippocampus of the mice. **a** Representative images of immunostaining for GABA, taken from the cell body layers in the dentate gyrus of a germ-free mouse and mice treated with *Lactobacillus reuteri*, *brevis*, or *plantarum*. Nuclei are shown in blue, GABA is shown in green. Using images covering the CA fields and the dentage gyrus, the percent GABA-positive cells was calculated by the fraction of cell bodies showing GABA (green color) from the total number of cells (identified by the blue nuclei). **b** and **c***Lactobacillus* inoculation (*n* = 4, 2 males and 2 females for each treatment) (**b**) or lactate treatment (*n* = 6, 3 males and 3 females for each treatment) (**c**) significantly increased the levels of GABA in the hippocampus of mice. Error bars indicate mean ± SEM

To further define the mechanism(s) underlying the improved memory response in mice following supplementation with *Lactobacillus* spp. or lactate, we quantified levels of GABA in the hippocampus proper, including the four Cornu Ammonis (CA) regions and the dentate gyrus (DG), using brain coronal sections taken from the treated mice. We chose to focus on the hippocampus as it is an essential area for the acquisition and formation of new memories, where GABA, the main inhibitory neurotransmitter in the brain, plays a critical role [24, 25]. As mentioned above, using metabolomic analysis we found that GABA levels were significantly higher in brain homogenates of mice inoculated with *L. plantarum*, but not with the other two species; although at a lower significance threshold GABA levels were also higher in the *L. reuteri* samples (*p* = 0.065; Fig. S3C). Using immunofluorescence, we quantified GABA expression in the hippocampus (Fig. S4) and GABA expression was compared between the GF controls and the three *Lactobacillus*-colonized mice groups (Fig. 5a, Fig. S4). All of the *Lactobacillus*-colonized mice showed higher fractions of cell bodies that expressed GABA in the hippocampus, compared to the germ-free control mice (Fig. 5b). However, our metabolite data showed no significant increases in GABA in stool or plasma samples of the inoculated mice compared to controls. Interestingly, we found that lactate treatment alone also increased the fractions of cell bodies that expressed GABA compared with control mice from 32% to 43% (*p* = 0.042; Fig. 5c), supporting the hypothesis that lactate could serve as the metabolic conduit between the gut and the brain.

A possible mechanism underlying the observed increase in GABA expression in the hippocampus could be an increase in the expression level of glutamate decarboxylase (GAD), which converts glutamate to GABA, in this brain area. To test this possibility, we quantified GAD_67_ gene expression levels in the dentate gyrus using single-molecule-based fluorescence in situ hybridization approach (fliFISH) [26]. However, we found no significant difference between GAD_67_ gene expression levels in germ-free mice and mice colonized with *Lactobacillus* strains (Fig. S5). Sequence analysis of the *Lactobacillus* isolates used in our study showed that *L. brevis* has two genes encoding glutamate decarboxylase and *L. plantarum* and *L. reuteri* each have one, suggesting that *Lactobacillus* may be the source of the increased GABA levels observed in the hippocampus. Another possible mechanism for the observed increase in GABA in the hippocampus of lactobacilli-inoculated mice is the non-oxidative metabolism and conversion of lactate to GABA via α-ketoglutarate transamination [27]. These hypotheses remain to be validated in future studies.

To further assess the translatable nature of our findings to the human host, co-culture experiments were performed with *L. reuteri* and human epithelial cells (Caco-2) using the HuMiX model (Fig. S6A). HuMiX is a microfluidics-based human-microbial co-culture system which was designed to simulate the environment of the human gastrointestinal tract where microbial and mammalian epithelial cells interact via soluble molecular factors [28]. The bacterial cells were inoculated after the epithelial cells had fully differentiated into polarized monolayers (after 7 days). Metabolites were measured in collected fluid samples from the bacterial and epithelial cell perfusion chambers for two time points (6 h and 24 h after incubation in addition to just before inoculation as baseline). Similar to the in vivo studies, we observed that lactate and mannitol increased over the incubation time (Figs. S6B-C and Table S7). We focused on lactate and found that it was present at significantly higher levels in both bacterial and perfusion chambers inoculated with *L. reuteri* compared to uninoculated controls (Fig. S6B). These results demonstrate that lactate produced by *L. reuteri* could diffuse to the opposite side of the chamber, indicating that it likely diffuses through the gut epithelial cell lining in humans and is transported to the brain via the bloodstream.

## Discussion

We conducted systematic genetic and microbial community profiling analyses to determine potential links between host genetics and the gut microbiome in memory. We demonstrated that members of the gut microbiome, specifically different *Lactobacillus* species, play a role in improving memory in mice. Also, we identified two sets of host genes, one previously associated with memory, cognition or other neurodevelopmental processes and the other set identified here as candidates for roles in cognition (Table S4). Examples of known human genes associated with memory also identified in our study include: *NTM* and *KLHl20* that are associated with Alzheimer’s disease and *DOCK8* and *KANK1* that are associated with neurodevelopmental disorders including memory potential [29–31]. Examples of known mouse genes associated with memory include *Specc1* that was identified in a mouse genetic screen for avoidance learning [32], *Prdx6* that was associated with neurogenesis and Alzheimer’s disease in mice [33, 34] and knock-out mice for *Abl2, Nrg3, Shank2, Gria1*, and *Fgf14* that caused neurodevelopmental defects [35–39]. The other set contains 135 genes not previously associated with memory of which 65 have brain expression [40] (http://connectivity.brain-map.org/). Additional experiments are required to assess their function in memory and to determine if and how they are linked to specific members of the gut microbiome.

We also provide metabolic evidence of links between the host, the gut microbiome and memory responses. Our untargeted approach in a complex microbial community-based system was able to identify significant correlations between memory and *Lactobacillus* within a background of the entire gut microbiome. Because we specifically identified *L. reuteri* in screening of the CC mouse cohort, we focused on that species, and included two other *Lactobacillus* species for comparison (*L. plantarum* and *L. brevis*). Subsequently, we confirmed that mono-association of gnotobiotic mice with any of the three *Lactobacillus* species was able to improve memory. This impact was not seen using *E. coli* as a negative control. This led us to hypothesize that lactobacilli produce specific metabolites that could cross the blood–brain barrier and influence memory of the host.

Because of recent reports which link specific *Lactobacillus* species and memory in mice (e.g., *L. rhamnosus* JB-1 [5], *L. helveticus* R0052 [6], *L. helveticus* NS8 [10]) and rats (e.g., *L. acidophilus* CUL60 and *L. acidophilus* CUL21 [8]), we focused on understanding the metabolic mechanism(s) underlying the memory improvement. Because we were interested in potential flow of metabolites from the gut, through plasma, to the brain, we examined samples from all three body locations for their metabolic compositions. Several metabolites were significantly higher in fecal samples of *Lactobacillus-*colonized mice, and less so in plasma and brain samples. In particular, in stool samples, there were specific metabolites that were significantly higher than GF controls for mice mono-associated with individual *Lactobacillus* strains, such as mannitol that was only observed for *L. reuteri* F275. In this case, mannitol injection did not improve memory over that of control mice. On the other hand, lactate was significantly higher in stool samples of mice inoculated with any one of the three lactobacilli and in brain samples with *L. plantarum* BDGP2 and trended towards higher levels in brain and plasma samples of mice inoculated with any one of the three species. Because lactate is produced by all lactobacilli and because it has previously been recognized as crucial for learning and long-term memory [41] we focused on lactate in subsequent targeted feeding experiments. Our results indicated that indeed lactate supplementation resulted in improvement in memory of CC042 mice that otherwise have a poor memory.

There are several clues in the literature towards the possible mechanism(s) underlying memory improvement with lactate. Suzuki et al. (2011) demonstrated that lactate derived from astrocytes via glycogenolysis was critical for long-term memory formation in rats [42]. Memory is an energy requiring process and the brain needs fuel to make and store memories. It is known that lactate is produced in astrocytes in the brain via glycolysis to provide energy to neurons [41]. Lactate administration by injection has previously been demonstrated to restore memory in chicks [43] and rats [42]. Lactate administration has also been found to enhance neuronal activity in vivo [44]. A recent study has shown that during exercise, lactate is released by the muscles, and crosses the blood–brain barrier to induce *Bdnf* expression and TRKB signaling in the hippocampus [45]. This enforces the hypothesis that a direct link from lactate to neurons exists that bypasses the need for glycogen energy storage in astrocytes [41]. While lactate has been implicated in several aspects of brain signaling, one of its roles might be metabolic [44]. Lactate has also been shown to signal changes in the NADH/NAD ratio [46] and lactate application increases intracellular levels of NADH [47]. Recent reviews have emphasized that the understanding of the role of lactate in memory is still in its infancy and there is the possibility that it plays several roles in this process [41, 48]. Also, we should note that other metabolites that we identified that were significantly higher in mice that were mono-associated with the different *Lactobacillus* species are potential targets for future study.

We also found an increase in GABA in the hippocampus of gnotobiotic mice that were mono-associated with either *L. reuteri* F275 *L. plantarum* BDGP2 or *L. brevis* BDGP6. These findings support previous findings of increased expression of GABA in the hippocampus of mice following treatment with *Lactobacillus rhamnosus* JB-1 [5, 12]. However, in older rats that were supplemented with a combined inoculum containing two strains of *Lactobacillus acidophilus* and two strains of *Bifidobacterium*, GABA increased in the frontal cortex, but not the hippocampus [8]. Recently, several gut microbes have been found to modulate GABA production pathways in the gut, suggesting a potential route for production of GABA that could serve to increase GABA levels in the brain [15]. Intriguingly, our finding that lactate treatment alone increased the fractions of cell bodies that expressed GABA compared with control mice (Fig. 4) supports the hypothesis that lactate could serve as a metabolic conduit for increased GABA production in the brain.

## Conclusion

Our study provides new evidence that links the complex genetic–microbiome-metabolome interplay that can contribute to memory. One potential outcome of this research is the support of use of probiotic *Lactobacillus* strains to promote memory through their production of lactate and through their promotion of GABA accumulation in the hippocampus, although it remains to translate these findings from mice to humans. In addition, our findings suggest that the metabolic mechanisms underlying the improved memory response by *Lactobacillus* in the diet could be at least partly due to the production of lactate in the colon that migrates through the blood to the brain.

## Methods

### Mice

All CC strains were purchased from the Systems Genetics Core Facility at the University of North Carolina (UNC). Passive avoidance memory test was assessed at 10–11 weeks of age. The study was carried out in strict accordance with the Guide for the Care and Use of Laboratory Animals of the National Institutes of Health. The Animal Welfare and Research Committee at Lawrence Berkeley National Laboratory approved the animal use protocol. Mice were maintained on PicoLab Rodent Diet 20 (5053), housed in standard micro-isolator cages on corn cobb bedding with enrichment consisting of crinkle cut, naturalistic paper strands. To test the effect of dietary lactate on memory, drinking water of CC042 mice was supplemented with sodium L-lactate (SIGMA; 71718) for 5 weeks starting at 4 weeks of age.

### Germ-free mice

Germ-free C57BL/6NTac mice were purchased from Taconic and were maintained within germ-free isolators. The status of our germ-free mice colony was tested every other week and after each opening of the transfer port. A sample was removed from the isolator consisting of fecal samples from multiple cages of animals housed in the isolator, water from the drinking bottles of all cages in the isolator and swabs of the inside of the cages, the floor, and entry port of the isolator. A portion of each sample was streaked onto a sheep blood agar plate and the swab was cultured in thioglycollate medium at 37 °C for 3 days, after which they were maintained at room temperature for 11 more days and observed for growth at 24-h intervals. In addition, every other month, samples were collected and tested for aerobic, anaerobic, and fungal growth at an independent commercial laboratory (IDEXX). All results for our animals were negative for bacterial and/or fungal growth.

### Mono-association of GF mice

At 3 weeks of age, GF mice were inoculated with either *Lactobacillus reuteri F275*, *L. plantarum* BDGP2 or *L. brevis* BDGP6 in the different isolators, which was confirmed by PCR and sequencing of their respective 16S rRNA genes. *Lactobacillus reuteri* F275 was purchased from ATCC (23272). *L. plantarum* BDGP2 [61] and *L. brevis* BDGP6 (unpublished; accession number CP024635) were isolated from *Drosophila* gut samples and verified by genome sequencing. *E.coli* strain DH10B was purchased from Invitrogen. GF mice were inoculated with 100 μl overnight cultures of *Lactobacillus* or *E.coli*.

### Passive avoidance memory test

Short-term memory of mice was assessed by passive avoidance using the Panlab passive avoidance box (Panlab: LE870/872). During the acquisition phase, mice were placed in the light compartment. When the mice innately crossed to the dark compartment, they received a mild foot shock (5 s; 0.3 mA). Duration of mice in the light compartment before entering to the dark compartment was recorded. Three days after the acquisition phase mice were again placed in the light compartment and the passive avoidance response was evaluated by measuring the latency to enter the dark compartment.

### QTL analysis of memory

Latency of entry into the dark compartment 3 days after the acquisition phase for all CC mice was used for genetic mapping. Genotype data for 134,593 SNPs was obtained from the UNC Systems Genetics Core website (http://csbio.unc.edu/CCstatus/index.py), and filtered for minor allele frequency > 5 out of the 29 CC strains, leaving 76,080 SNPs. At each SNP, latency to enter on day 3 for all CC mice were assigned to their respective alleles. We then used Mann–Whitney U to test the significance of associations between memory and allele classes at each SNP.

### Microbiome analyses

Genomic DNA was extracted from the homogenized fecal samples using the PowerSoil DNA Isolation Kit (http://www.mobio.com/) according to the manufacturer’s instructions. PCR amplification of the V4 region of the 16S rRNA gene was performed using the protocol developed by the Earth Microbiome Project (http://press.igsb.anl.gov/earthmicrobiome/empstandard-protocols/16s/) and modern primers [49]. Amplicons were sequenced on an Illumina MiSeq using paired, 250 base-pair reads, according to the manufacturer’s instructions and are available on OSF (https://osf.io/jbt5g/). The Hundo amplicon processing protocol was used to process 16S and ITS amplicons [50]. In brief, sequences were trimmed and filtered of adapters and contaminants using BBDuk2 of the BBTools package. VSEARCH [51] was used to merge, filter to an expected error rate of 1, dereplicate, and remove singletons before preclustering reads for de novo and reference-based chimera checking. Reads were clustered into OTUs at 97% similarity and an OTU table in the BIOM format [52] was constructed by mapping filtered reads back to these clusters. BLAST+ [53] is used to align OTU sequences to the database curated by CREST [54] (SILVA v128 for 16S) and taxonomy was assigned based on the CREST LCA method. Graphing was performed in R, making use of the Phyloseq package [55].

*L. plantarum* BDGP2 [56] and *L. brevis* BDGP6 (unpublished) were sequenced using the PacBio long read strategy. After assembly the genomes were annotated for predicted protein-coding open reading frames using Rapid Annotation of microbial genomes using Subsystems Technology tool [57] and the GenBank annotation pipeline. The RAST and GenBank produced gene models for *L. plantarum*, *L. brevis*, and *L. reuteri* predict: six L-lactate dehydrogenase (EC 1.1.1.27) encoding genes, one D-lactate dehydrogenase (EC 1.1.1.28) encoding gene and one Glutamate decarboxylase (EC 4.1.1.15) encoding gene; two L-lactate dehydrogenase encoding genes, two D-lactate dehydrogenase encoding genes, and two Glutamate decarboxylase encoding genes; and five L-lactate dehydrogenase encoding genes, one D-lactate dehydrogenase encoding gene, and one Glutamate decarboxylase encoding gene, respectively.

### Metabolome analyses

Metabolites were extracted from mouse fecal, plasma, and whole brain homogenate samples. Fecal samples were extracted with methanol as reported previously [21], and plasma and whole brain homogenates were extracted using the MLPEx method [58]. Briefly, 50 μL of plasma was extracted with 200 μL of chloroform/methanol (2:1, *v*/*v*), and extracted molecules in both aqueous and organic layers were combined and dried in vacuo. Whole brains were weighed and extracted using MPLEx, but the volume of solvent was added proportionally to the amount of tissue. All the extracts were stored at − 80 °C, and they were analyzed by gas chromatography coupled to mass spectrometry as reported previously [21]. All the raw MS data files are available at the OSF public data depository (https://osf.io/jbt5g/).

### Immunostaining of GABA in mouse hippocampus

Mice brains were fixed in 4% paraformaldehyde and embedded in paraffin. Two male and two female mice were analyzed for each treatment (*n* = 4). Coronal sections were generated and placed on class slides. The sections were rehydrated by first immersing the slides in xylene (mixed isomers) for 20 min. The slides where then incubated in 100% ethanol for 20 min, followed by incubations in ethanol at decreasing concentrations (100%, 95%, 70%, and 50%), for 5 min each. The slides where then rinsed using deionized water. Heat-induced epitope retrieval was done by 5 min incubation of the slides in 10 mM Tris, 1 mM EDTA heated to 95 °C, followed by rinsing in water. Immunostaining of the hippocampus proper, including the Cornu Ammonis (CA) fields and the dentate gyrus (DG), was done using anti-GABA antibody produced in rabbit (Sigma A2052) and applied to the sections at 1:200 dilution, followed by a fluorescent (Alexa488) Goat anti-rabbit IgG H+L (ThermoFisher A11008) applied at 1:400 dilution. Antibody dilutions were made in PBS containing 0.25% Triton X-100 and 5% goat serum. The sections were then stained with DAPI (1 μg/ml in PBS for 10 min) and washed with PBS. Vectashield drops were placed on the sections and glass coverslips were placed on top of them and sealed using nail polish. Fluorescence imaging was done using inverted confocal fluorescence microscope (Zeiss). Initially, 10 × objective and tiling over the whole section was used, followed by imaging the hippocampus, including the CA fields and DG, using 40 × water immersion objective to achieve the magnification needed for identifying and counting cell bodies (Fig. S4). GABA expression was quantified by calculating the number of cell bodies showing GABA expression (green) as the percent of all cell bodies, detected by the DAPI-stained nuclei (blue). Significant differences between germ-free and *Lactobacillus*-treated mice were determined using *t* tests.

### Glutamate decarboxylase (GAD_67_) gene expression using fliFISH in mouse brain sections

Fluctuation localization imaging-based FISH (fliFISH) was developed and performed as described in Cui et al. [26]. In short, fliFISH utilizes photoswitchable dyes and super-resolution localization microscopy to accurately count and localize mRNA molecules with a small number of oligonucleotide probes. The single-molecule on-time fraction (*F*_single_) for Alexa647 was found to be 0.2% under 0.5 kW/cm^2^ excitation. When using 20 probes (*n*), each tagged with one dye molecule, to target a transcript, the ensemble on-time fraction (*F*_ensemble_*= 1−(1−F*_single_*)*^*n*^) of 4% should be detected from a successfully hybridized RNA transcript position. In contrast, stray or nonspecifically bound probes would generate roughly a single-molecule on-time fraction values, while strong autofluorescence and aggregated probes would generate higher ensemble on-time fraction values. In addition, fliFISH enables to resolve multiple transcripts in a diffraction-limited area as the centroid of each blinking event is registered with 15–25 nm resolution in the super-resolution reconstructed image.

The 24 primary FISH probes used in this study were designed to have two segments: a GAD_67_ transcript targeting domain, and a terminal overhang. The targeting domain was generally 20 nucleotide-long, with 45–55% CG content, no self-repeats and inner loop-stem structures. The secondary probe was labeled with two Alexa647 dye molecule, one in each end, and was designed to hybridize with the overhang sequence. All the probe sequences were subjected to BLAST searching to avoid nonspecific targeting and purchased from Integrated DNA Technologies.

The hybridization procedure followed previously established protocols [59, 60]. Primary probes were mixed and hybridized with the secondary probe to form fluorescent complexes before introducing to the tissue sections following a published protocol [60]. One microliter from each of the 24 oligonucleotide probes 100 μM solutions was mixed together and water was added to a total of 120 μl. Six microliters were then taken out and mixed with 1.5 μl 100 μM secondary probe, 2 μl 10 × NEB3 buffer (containing 1 M NaCl, 0.5 M Tris-HCl, 0.1 M MgCl_2_), and 10.5 μl water. The mixture was heated to 85 °C and gradually cooled down to room temperature. The microscope slides with paraffin-embedded mouse brain sections were dipped in 100% xylenes for 10 min, followed by100%, 95%, and 70% ethanol for 10 min each. The slides were then left in 70% ethanol overnight at 4 °C and washed with PBS before hybridization. Hybridization was done by first washing the slides with “wash buffer” (containing 2 × SSC and 10% formamide). 200 μl “hybridization buffer” (containing 10% dextran sulfate, 2 × SSC and 10% formamide) were mixed with 3.3 μl of the FISH probe solution. Drops of this mixture were placed on the tissue sections and the slides were kept overnight at 46 °C. The slides were then washed twice by incubating in “wash buffer” for 20 min at 46 °C, followed by a wash with 2 × SSC buffer. The slides were then incubated in 1 μg/ml DAPI solution for 10 min and were washed using PBS.

The fliFISH images were taken using a home-built, Zeiss Axioobserver based single-molecule imaging system. A 100 × oil immersion objective lens (NA 1.4, Plan Apo) and an EMCCD camera (Andor iXon Ultra 897) were used. DIC and DAPI fluorescence images were taken in addition to fluorescent Alexa 647 single molecules images. Over 10,000 image frames were taken (at 25 Hz frame rate) for post-processing. Gaussian musk fitting algorithm was applied to find the central location of each emission event and nearby events were grouped together (assigned to the same transcript) using the DBSCAN algorithm. For the grouped emission events, the center of mass was determined to represent the possible existence of a transcript. All image processing was performed with MATLAB and C scripts that are available upon request.

### HuMiX-based analyses

HuMiX-based co-cultures involving *L. reuteri* and the human epithelial cell line Caco-2 were performed as previously described [28]. Caco-2 were allowed to fully differentiate for 7 days at which point the co-cultures with *L. reuteri* were established. Eluates were collected from the microbial and perfusion chambers just before inoculation as well as after 6 and 24 h of co-culture and immediately flash-frozen for subsequent metabolomic analyses.

### Statistics

To evaluate the association between the memory latency to enter time and the microbiome features, we employed Cox Proportional-Hazards Regression analysis, where both forward and backward feature selection strategies were used to optimize the subset of microbes that significantly impact memory. During forward selection, each individual microbe at the OTU level was evaluated through the Cox Proportional-Hazards Regression analysis, and only those with significant impact on memory (round (*P* value, 2) ≤ 0.05, where round (*X*,*N*) rounds *X* to its nearest *N* decimal digits) were selected (6 OTUs: f__Anaeroplasmataceae, f__Bacteroidaceae, f__Bacteroidales_S24-7_group, f__Lactobacillaceae, f__Deferribacteraceae, f__Clostridiaceae_1). During backward selection, the combined subset of features (multivariates) were further evaluated through the Cox Proportional-Hazards Regression analysis, where only the features with significant impact (round (*P* value, 2) ≤_0.05) were retained. Different from forward selection, the backward selection was performed in an iterative manner until all the features in the refined subset were significantly associated with memory. In our study, the backward selection with 2 iterations led to the refined final subset of four microbiome features (i.e., f__Bacteroidaceae, f__Lactobacillaceae, f__Deferribacteraceae, and f__Clostridiaceae_1), where f__Bacteroidaceae and f__Clostridiaceae_1 are “prognostic” unfavorable (hazard ratio >_1), and f__Lactobacillaceae and f__Deferribacteraceae are “prognostic” favorable (hazard ratio <_1).

Differences in memory potential between germ-free and *Lactobacillus* inoculated mice or between lactate treated and control mice was assessed by non-parametric test (Mann–Whitney test). Difference in metabolite abundance and GABA expression between germ-free and *Lactobacillus* inoculated mice was assessed by Student’s *t* test. Significance was determined at *P*_<_0.05. Multivariate analysis of variance (MANOVA) was used to examine for statistical differences in fecal metabolite profiles across germ-free and *Lactobacillus*-inoculated mice.

## Supplementary information

**Additional file 1.**

## Data Availability

Mouse gut microbiome 16S rRNA gene sequence data is available on OSF (https://osf.io/jbt5g/). All the raw metabolomic data are available at the OSF public data depository (https://osf.io/jbt5g/).

## Abbreviations

CC: Collaborative Cross
GF: Germ-free
GWAS: Genome-wide Association Analysis
SNP: Single-nucleotide polymorphisms
GABA: Gamma-aminobutyric acid
GAD: Glutamate decarboxylase
fliFISH: Single molecule-based fluorescence in situ hybridization
MANOVA: Multivariate analysis of variance
