# Correction to: Genetic and metabolic links between the murine microbiome and memory

**DOI:** 10.1186/s40168-020-00870-5

**Published:** 2020-05-28

**Authors:** Jian-Hua Mao, Young-Mo Kim, Yan-Xia Zhou, Dehong Hu, Chenhan Zhong, Hang Chang, Colin J. Brislawn, Sarah Fansler, Sasha Langley, Yunshan Wang, B. Y. Loulou Peisl, Susan E. Celniker, David W. Threadgill, Paul Wilmes, Galya Orr, Thomas O. Metz, Janet K. Jansson, Antoine M. Snijders

**Affiliations:** 1 Biological Systems and Engineering Division, Lawrence Berkeley National Laboratory, Berkeley, CA 94720 USA; 2 Earth and Biological Sciences Directorate, Pacific Northwest National Laboratory, Richland, WA USA; 3 Marine College, Shandong University, Weihai, 264209 China; 4 Department of Clinical Laboratory, The Second Hospital of Shandong University, Jinan, 250033 Shandong China; 5 Luxembourg Centre for Systems Biomedicine, University of Luxembourg, 7, Avenue des Hauts Fourneaux, L-4362 Esch-sur-Alzette, Luxembourg; 6 Department of Veterinary Pathobiology, A&M University, College Station, Texas USA; 7 Department of Molecular and Cellular Medicine Texas, A&M University, College Station, Texas USA

**Correction to: Microbiome (2020) 8:53**

**https://doi.org/10.1186/s40168-020-00817-w**

Following publication of the original article [1], the authors reported that Sarah Fansler had been left off the author list in error. The author group has been updated above and the original article [1] has been corrected.
