# Supplementary material for: Genetic and metabolic links between the murine microbiome and memory
Source: Microbiome. 2020 Apr 16;8:53. doi: 10.1186/s40168-020-00817-w (PMC7164142; doi:10.1186/s40168-020-00817-w)
Supplement: Supplementary file 2 — Additional file 1. [file 40168_2020_817_MOESM1_ESM.zip › 40168_2020_817_MOESM1_ESM.pdf]

Supplementary Materials

Genetic and metabolic links between the murine microbiome and memory

Jian-Hua Mao[1]*, Young-Mo Kim[2]*, Yan-Xia Zhou[1,3]*, Dehong Hu[2], Chenhan Zhong[1], Hang Chang[1], Colin Brislawn[2], Sasha Langley[1], Yunshan Wang[1,4], B.Y. Loulou Peisl[5], Susan E. Celniker[1], David W. Threadgill[6,7], Paul Wilmes[5], Galya Orr[2], Thomas O. Metz[2], Janet K. Jansson[2#] and Antoine M. Snijders[1#]

Supplementary Tables

Table S1. Passive avoidance test results in CC mice.
See separate excel sheet.

Table S2. Genome wide genetic association of the memory phenotype.
See separate excel sheet.

Table S3. Enrichment analysis of 222 genes located in genetic loci significantly associated with memory.
See separate excel sheet.

Table S4. Candidate genes located in genetic loci significantly associated with memory.
See separate excel sheet.

Table S5. List of OTUs and taxonomic assignments based on 16S sequencing of mouse fecal samples.
See separate excel sheet.

Table S6. Fecal metabolite abundance levels in *Lactobacillus* colonized and germ-free mice.
See separate excel sheet.

Table S7. Metabolite abundance levels in Humix chamber before and after *Lactobacillus* colonization.
See separate excel sheet.

Supplementary Figures 1 - 6

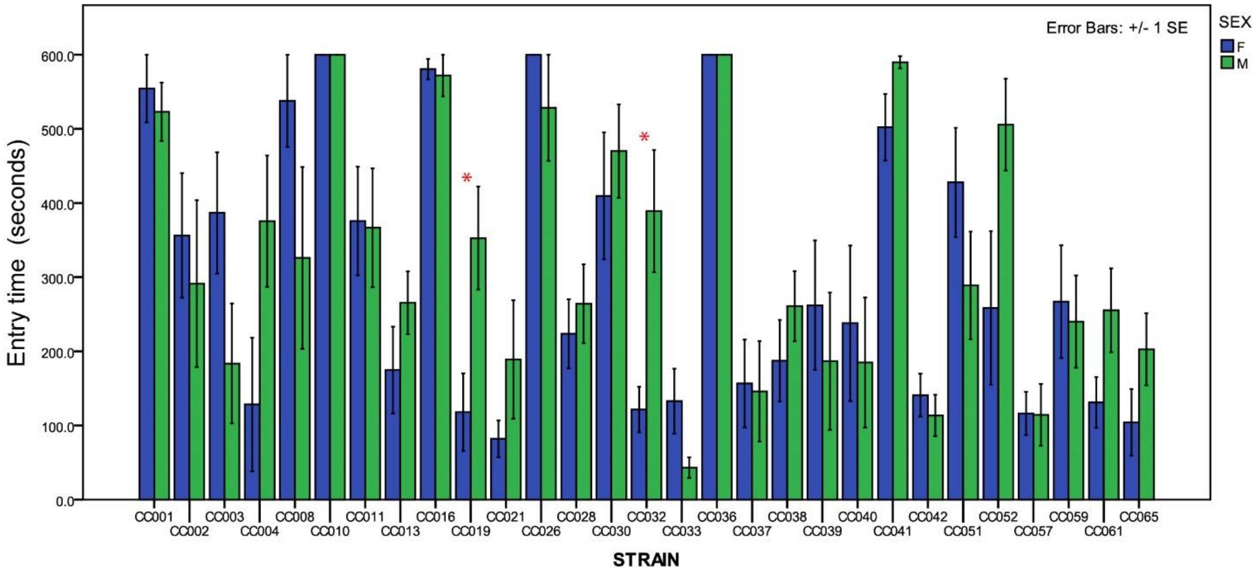

Figure S1. Sex differences in memory potential across CC strains. Red asterisk indicates significant difference between male and female mice (p<0.05 by non-parametric test.

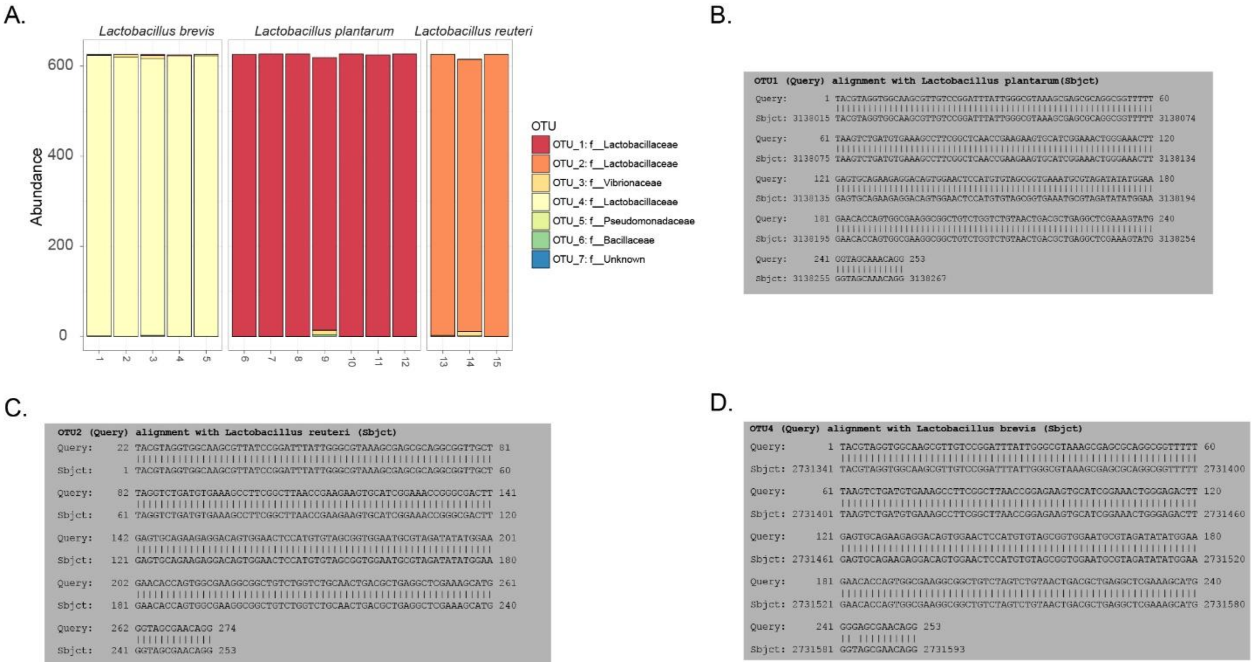

Figure S2. Gut microbiome composition confirmation of *Lactobacillus* colonized germ-free mice by 16S sequencing. Within our three inoculated groups we identified three Lactobacillus OTUs (OTU1, OTU2 and OTU4). Each OTU was dominantly present in a single treatment group and could be traced to the inoculated species.

A.

FECAL

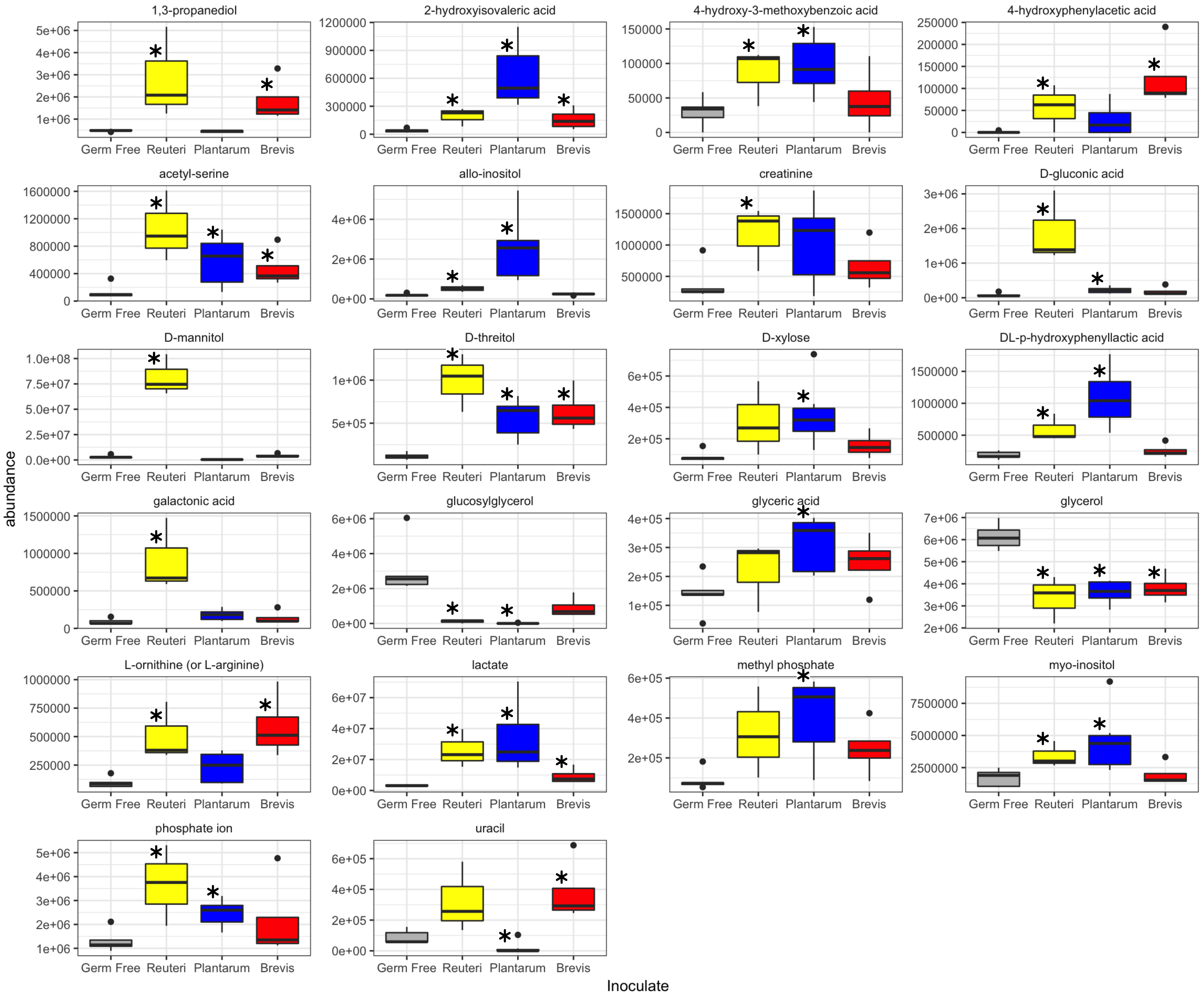

Figure S3. Metabolome analysis of fecal, plasma and brain samples of Lactobacillus colonized germ-free mice. Relative abundance level of metabolites in fecal (A), plasma (B) and brain (C) samples from germ-free and Lactobacillus inoculated mice.

Figure S3 continued

B.

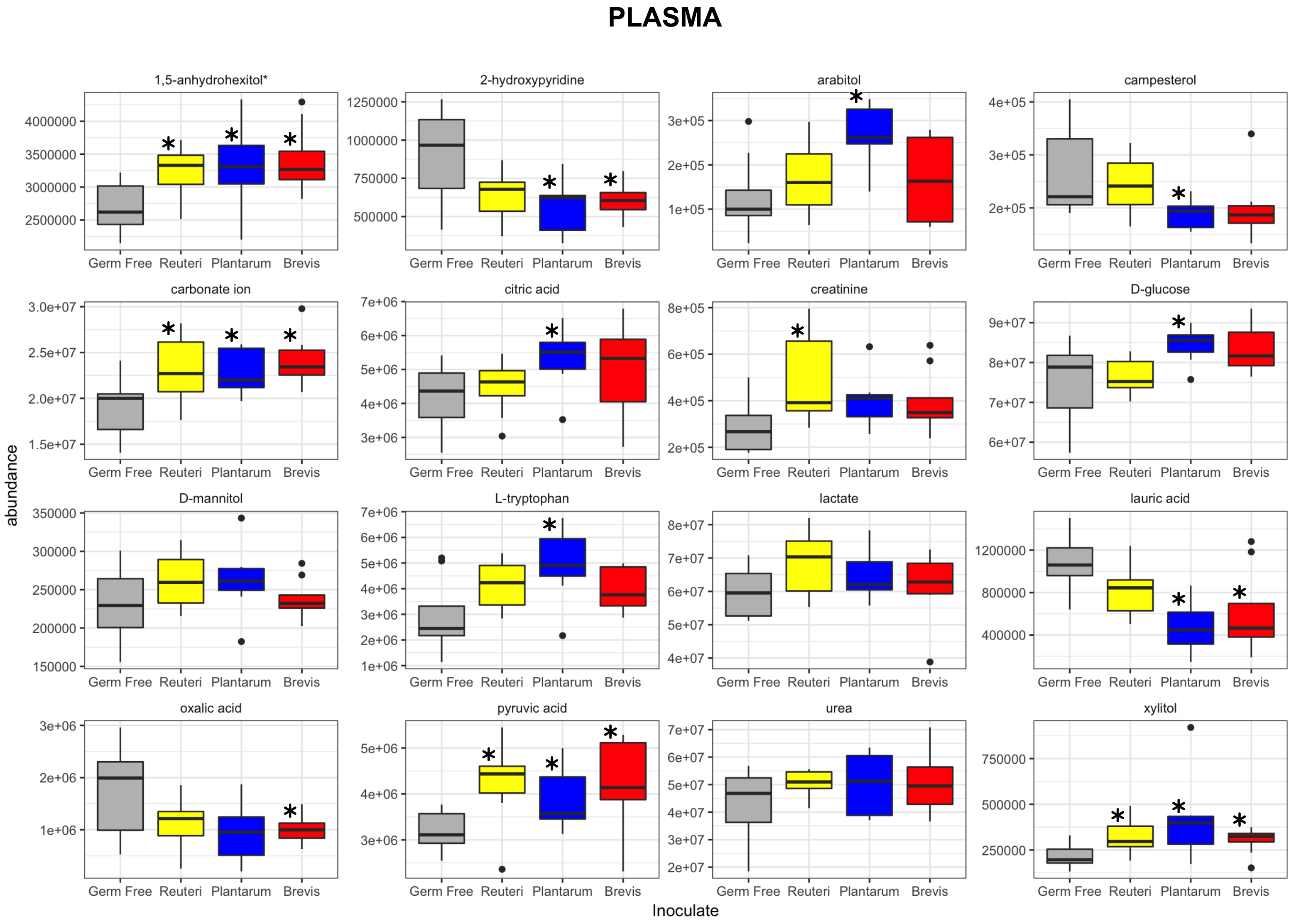

Figure S3 continued

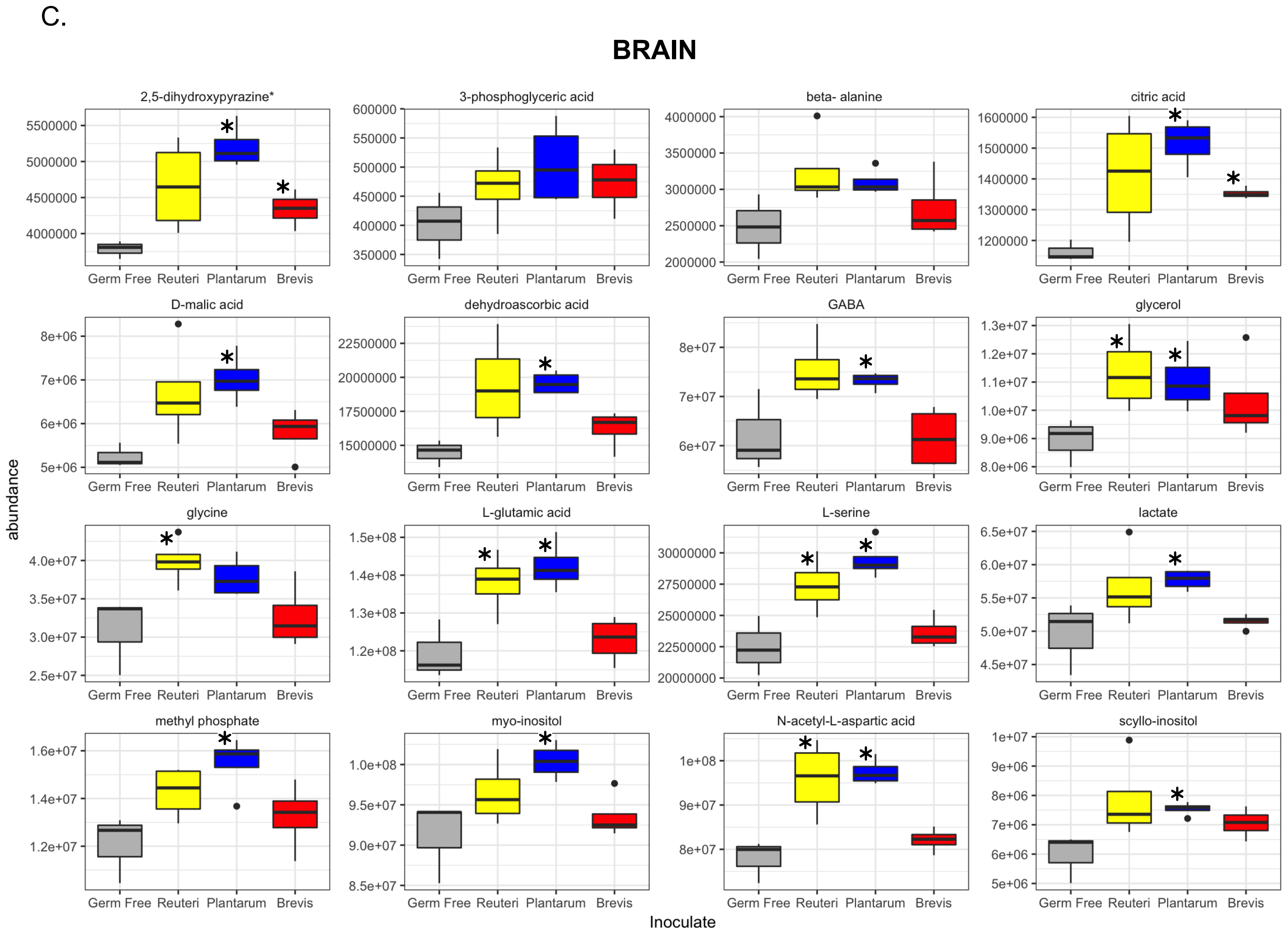

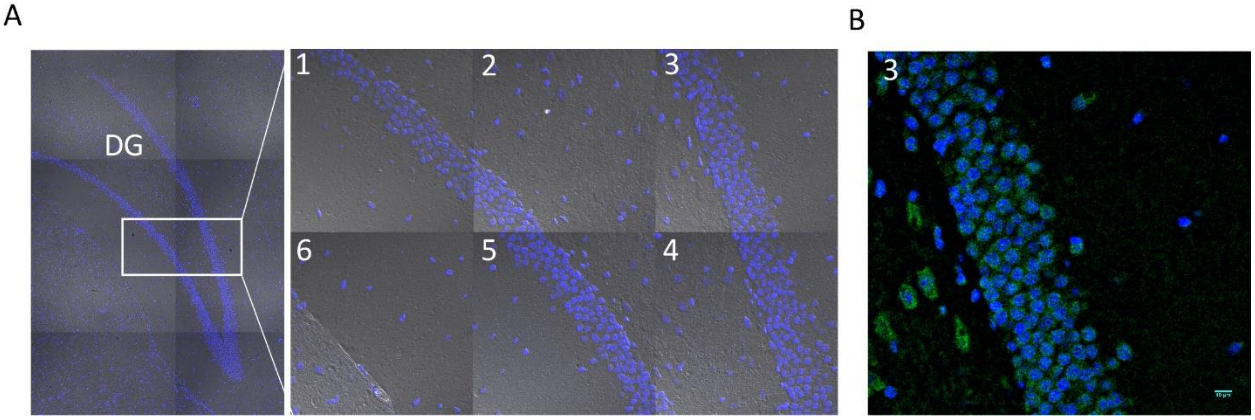

Figure S4. Expression levels of GABA in hippocampus of germ-free mice and mice colonized with *lactobacillus* strains.
A. A representative image of the dentate gyrus (DG) taken from a coronal brain section of a mouse colonized with *Lactobacillus reuteri*, where the nuclei are stained in blue. Individual adjacent images covering the main hippocampal regions, including the CA1-4 fields and the DG, were taken using a 10X objective and stitched together. The hippocampal area was then divided to smaller areas in the size of the white rectangle, which were imaged again with 40X objective to generate 6 images each, numbered 1-6. B. Each of the numbered images (from A) was then used to count both the total number of nuclei, and those nuclei surrounded with GABA (green), detected by immunofluorescence. Image #3 is shown here as an example. The percent cell-bodies expressing GABA was then calculated as the fraction of the total number of nuclei.

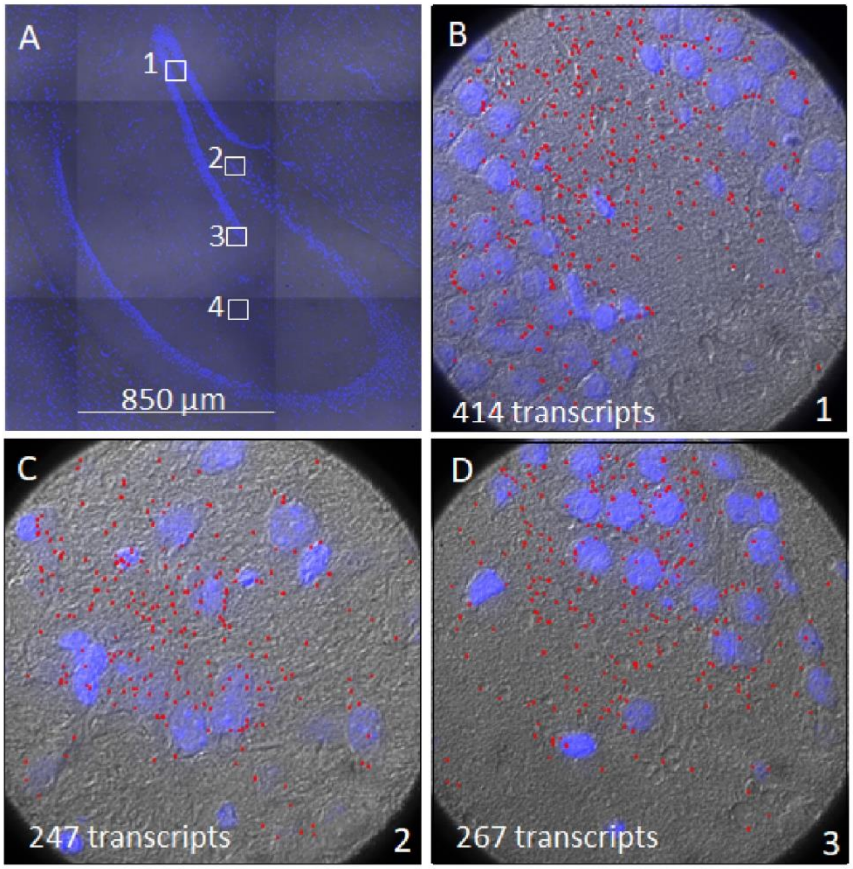

Figure S5. Expression levels of glutamate decarboxylase (GAD$_{67}$) in the dentate gyrus of germ-free mice and mice colonized with different *Lactobacillus* strains.
A. A representative image of the hippocampus taken from a coronal brain section from a mouse colonized with *Lactobacillus plantarum*, showing four 83 x 83 µm areas (white squares) that were used for counting GAD67 transcripts. Nuclei are stained in blue.
B-D. The overlaid fluorescence and DIC images in B, C and D are high magnification images of three of the four small areas, marked as 1, 2 and 3 in A, respectively. Fluctuation localization imaging-based fluorescence in situ hybridization (fliFISH) was used to count the number of transcripts (red dots) in each small area. Two male and two female mice were used for each treatment group. No significant difference was found between *Lactobacillus* colonized mice and GF mice when comparing transcript counts between similar areas or when comparing the sum of transcripts in the four areas.

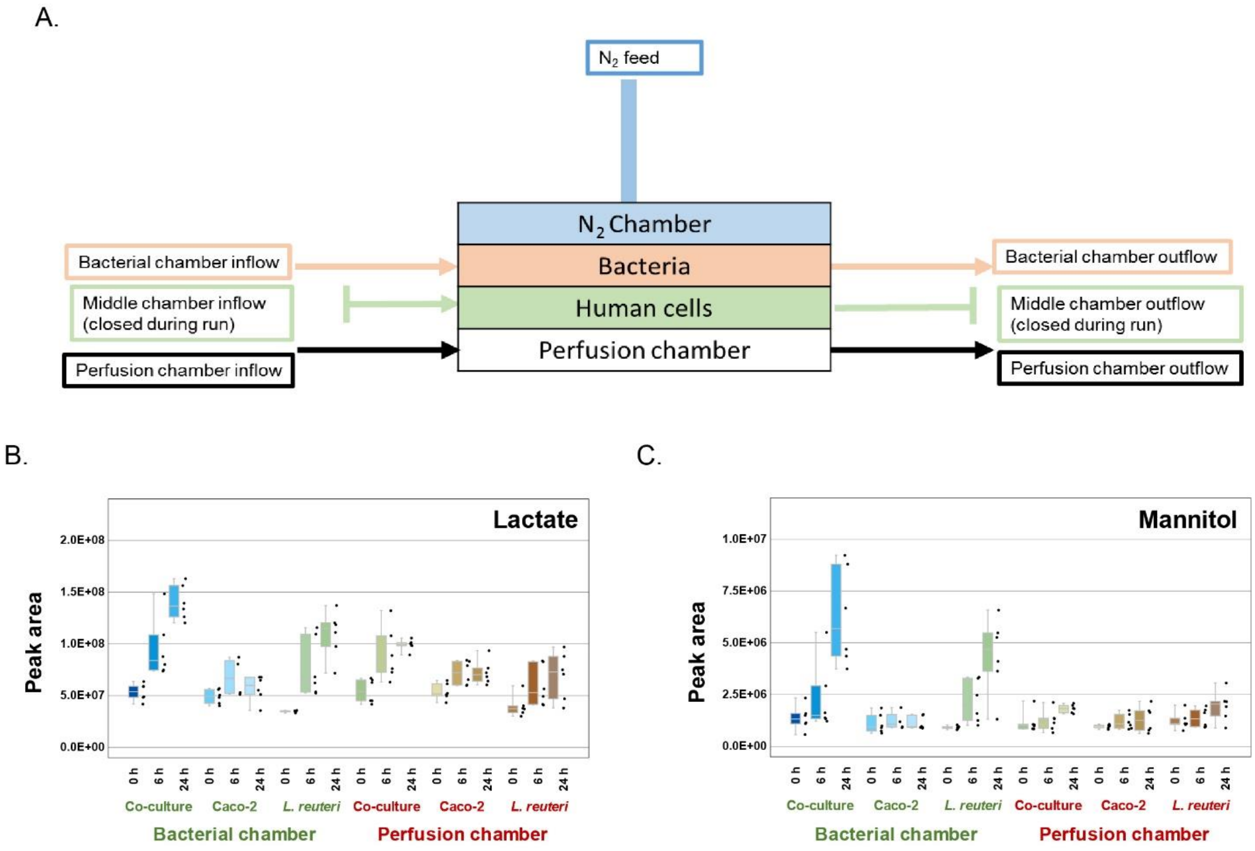

Figure S6. Metabolite profiling of eluates from the HuMiX model.
A. Schematic of the HuMiX model. The middle chamber (human cells) is closed during the experiment and the human epithelial cells are supplied with media from the basal side via diffusion from the perfusion chamber. The eluates from the bacterial chamber and perfusion chamber outflow was collected at different timepoints (before bacteria inoculation, 6 and 24 h after inoculation). B. Lactate levels in bacterial and perfusion chamber eluates at three different timepoints. C. Mannitol levels in bacterial and perfusion chamber eluates at three different timepoints.
